# Supplementary material for: De novo and scaffold-based design of GDF15 binders for cancer cachexia diagnostics and therapeutics
Source: Exp Mol Med. 2026 May 8;58(5):1626–41. doi: 10.1038/s12276-026-01727-x (PMC13234300; doi:10.1038/s12276-026-01727-x)
Supplement: Supplementary file 1 — Supplementary Information [file 12276_2026_1727_MOESM1_ESM.pdf]

Supplementary Information

De novo and scaffold-based design of GDF15 binders for cancer cachexia diagnostics and therapeutics

Jinsook Ahn[1,*], Ryeongeun Cho[2,*], Sohyun Kim[3], Dongsun Lee[1], and Ho Min Kim[1, 2, 3]

This PDF file includes :

Supplementary Figs. 1 to 6

Supplementary Table. 1

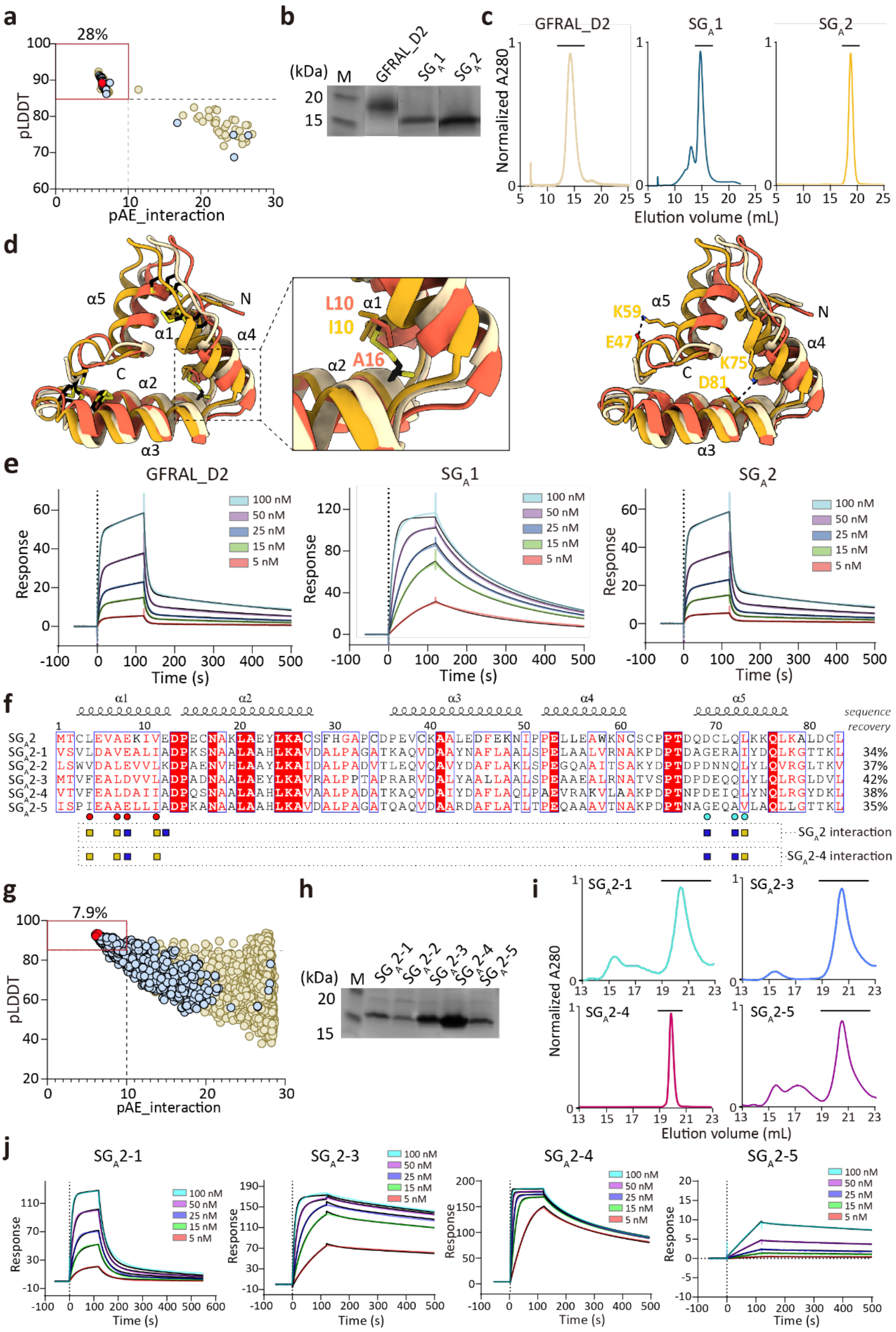

Supplementary Fig. 1. Structural and biochemical characterization of scaffold-grafted GDF15 site A binders.

a, g. Filtering metrics for initial GFRAL D2–based sequence designs **(a)** and scaffold-guided partial diffusion variants **(g)**. Candidates meeting the criteria of pLDDT > 85, pAE_interaction < 10, and ΔΔG < –30 are enclosed in red boxes. Overall,

28% of designs (28%, 28/100) from the first sequence design **(a)** and 7.9% from the partial diffusion designs (7.9%, 306/3850) **(g)** passed the filtering criteria. Data points with ΔΔG < –30 are shown in blue; those with ΔΔG > –30 are shown in yellow. Among these, final candidates selected for experimental validation (protein expression, purification, and binding analysis) are indicated with red circles.

b, h. SDS-PAGE analysis of purified binders: **(b)** SG$_{A}$1 and SG$_{A}$2; **(h)** SG$_{A}$2-1 to SG$_{A}$2-5, after *E. coli* expression and affinity purification.

c, i. SEC profiles of designed binders: **(c)** GFRAL D2, SG$_{A}$1, and SG$_{A}$2; **(i)** SG$_{A}$2-1, SG$_{A}$2-3, SG$_{A}$2-4, and SG$_{A}$2-5.

d. Structural alignment of GFRAL D2 (beige), SG$_{A}$1 (orange), and SG$_{A}$2 (yellow), showing overall fold similarity with subtle structural differences. Residue substitutions at C10 and C16 (C10L/C16A in SG$_{A}$1 and C10I in SG$_{A}$2) disrupt the disulfide bond (left). In SG$_{A}$2, additional substitutions create new interactions between E47–K59 and K75–D81 that shift α3 and α5 (right).

e, j. SPR analysis of binding to recombinant GDF15 dimer: GFRAL D2, SG$_{A}$1, and SG$_{A}$2 **(e)**; SG$_{A}$2-1, SG$_{A}$2-3, SG$_{A}$2-4, and SG$_{A}$2-5 **(j)**. Sensorgrams are shown for analytes ranging from 5 to 100 nM (indicated binder).

f. Sequence alignment of SG$_{A}$2 with scaffold-guided partial diffusion designs (SG$_{A}$2-1 to SG$_{A}$2-5). Key interacting residues conserved in both SG$_{A}$2 and SG$_{A}$2-4 are marked with cyan circles, while residues contributing to affinity differences between the two binders are highlighted with red circles. Sequence Recovery (SR) values, calculated based on the percentage identity to the SG$_{A}$2 reference sequence, are indicated to the right of each alignment. Key hydrophobic (yellow square) and electrostatic (blue square) residues interacting with GDF15 are marked.

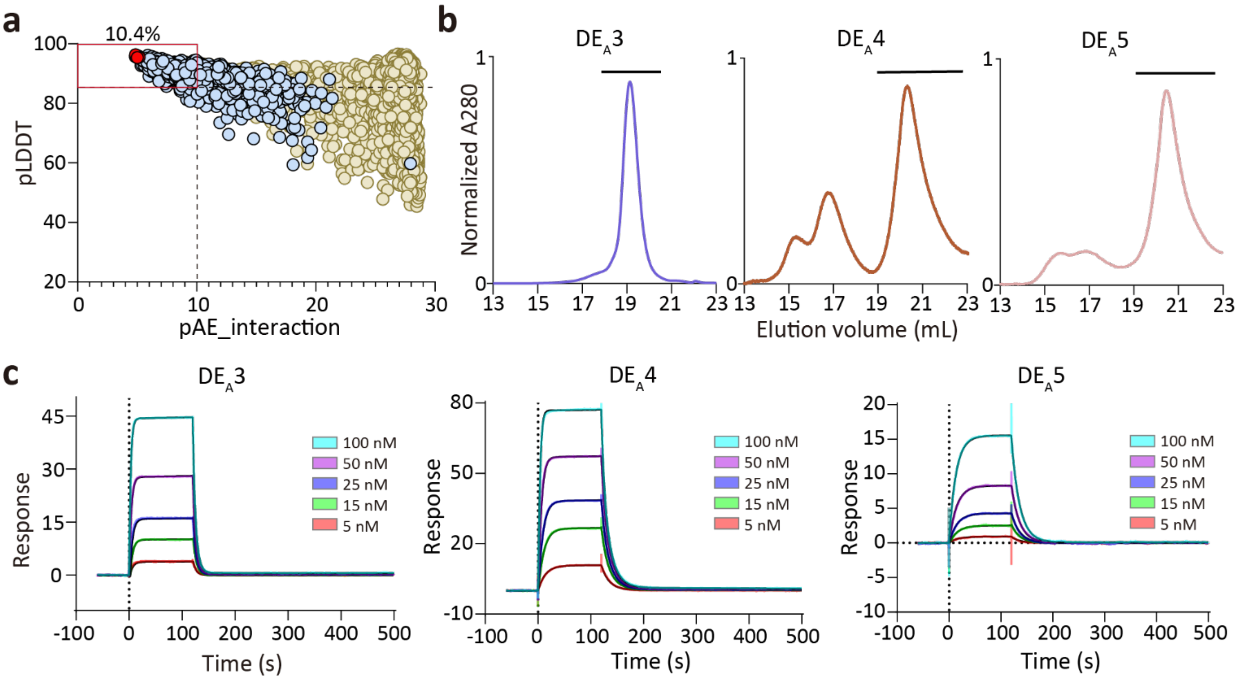

Supplementary Fig. 2. Structural and biochemical characterization of *de novo* designed GDF15 site A binders.

a. Filtering metrics for *de novo*–designed binders using RFdiffusion and ProteinMPNN. Candidates with pLDDT > 85, pAE_interaction < 10, and ΔΔG < –30 were selected (red box). Overall, 10.4% of the total designs (10.4%, 539/5,184) passed the filtering criteria. Data points with ΔΔG < –30 are shown in blue; those with ΔΔG > –30 are shown in yellow. Among these, final candidates selected for experimental validation (protein expression, purification, and binding analysis) are indicated with red circles.

b. SEC profiles of *de novo* designed binders (DE$_A$3 to DE$_A$5).

c. SPR analysis of DE$_A$3, DE$_A$4, and DE$_A$5 binding to recombinant GDF15 dimer. Sensorgrams are shown for analytes ranging from 5 to 100 nM (indicated binder).

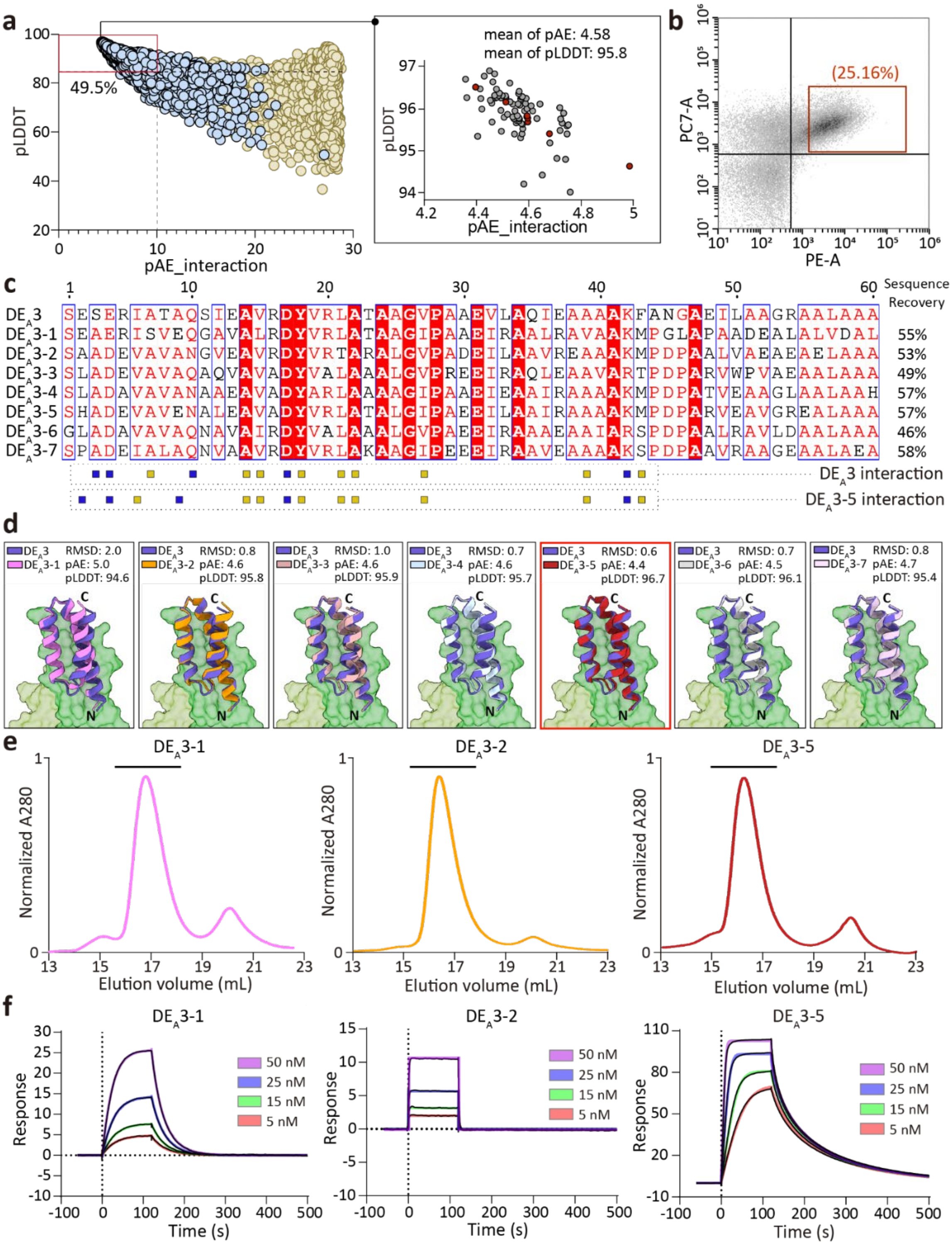

Supplementary Fig. 3. Structural and biochemical characterization of DE$_A$3-derived GDF15 site A binders.

a. Filtering metrics of DE$_A$3-guided partial diffusion variants. Candidates with pLDDT > 85, pAE_interaction < 10, and ΔΔG < –30 were selected (red box). Overall, 49.5% of the total designs (49.5%, 3775/7620) passed the filtering criteria. Data points with ΔΔG < –30 are shown in blue; those with ΔΔG > –30 are shown in yellow. Among these, final candidates selected for experimental validation (protein expression, purification, and binding analysis) are indicated by red circles. The top 100 candidates indicated in the black box are screened by yeast surface display.

b. Yeast surface display screening and FACS-based enrichment of GDF15 binders. In the two-dimensional FACS plot, the x-axis shows GDF15 binding (PE fluorescence) and the y-axis indicates surface displayed binder (PE-Cy7). High-expression and high-binding clones (upper right quadrant, 25.2% of the total population) were isolated for further analysis.

c. Sequence alignment of the parental scaffold DE$_A$3 and the partial diffused binders (DE$_A$3-1 to DE$_A$3-7). Key hydrophobic (yellow square) and electrostatic (blue square) residues interacting with GDF15 are marked.

d. Structural alignment of DE$_A$3 with DE$_A$3-derived binder variants. RMSD with AF2-predicted structure and AF2 scores (pAE_interaction and pLDDT) for the binder/GDF15 complex are indicated.

e. SEC profiles of DE$_A$3-derived binders (DE$_A$3-1, DE$_A$3-2, and DE$_A$3-5).

f. SPR analysis of DE$_A$3-1, DE$_A$3-2 and DE$_A$3-5 binding to recombinant GDF15 dimers. Sensorgrams are shown for 5-50 nM analytes (indicated binder).

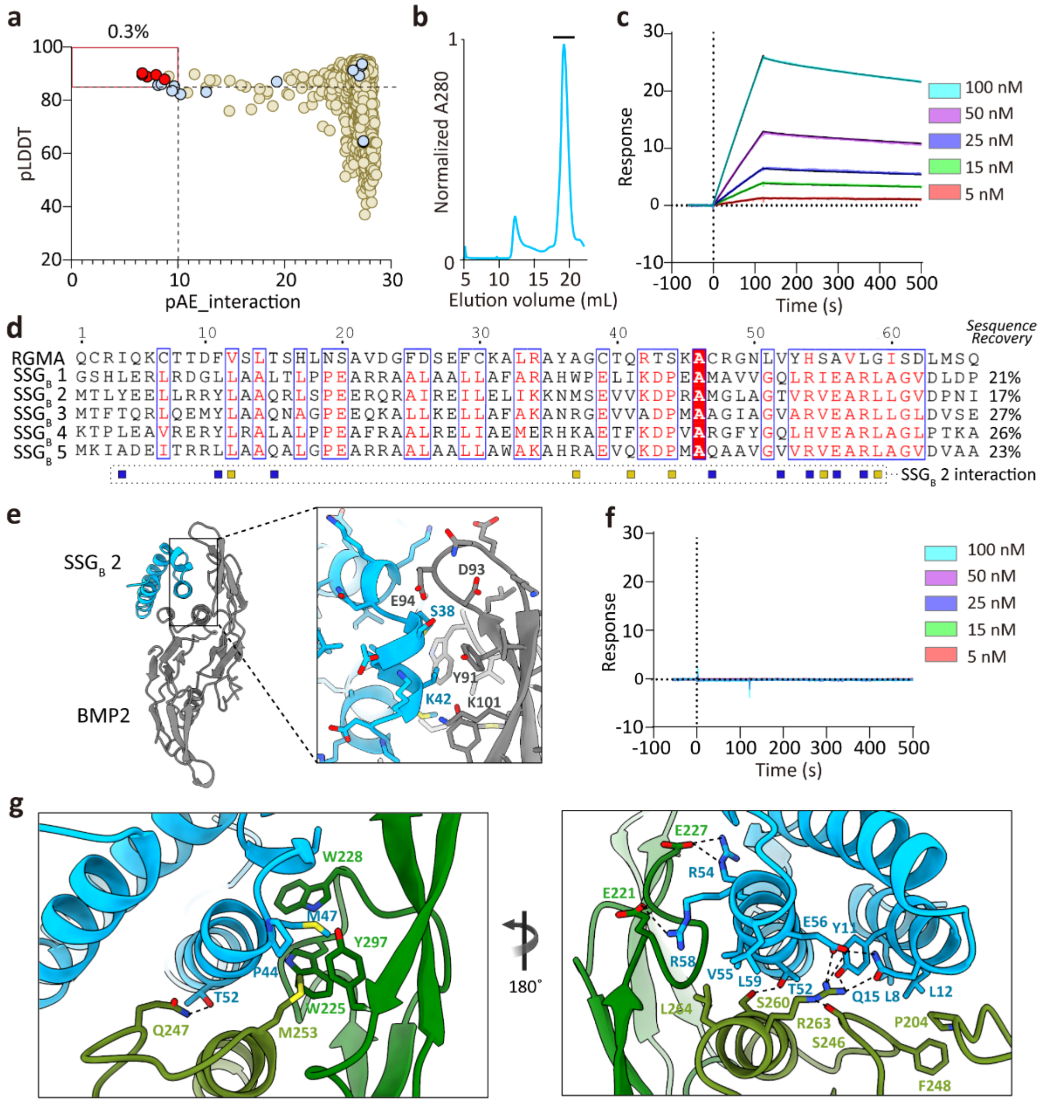

Supplementary Fig. 4. Structural and biochemical characterization of SSG-designed GDF15 site B binders.

a. Filtering metrics of RGMA-guided partial diffusion variants. Candidates with pLDDT > 85, pAE_interaction < 10, and ΔΔG < –30 were selected (red box). Overall, 0.3% of the total designs (0.3%, 10/3000) passed the filtering criteria. Data points with ΔΔG < –30 are shown in blue; those with ΔΔG > –30 are shown in yellow. Among these, final candidates selected for experimental validation (protein expression, purification, and binding analysis) are indicated by red circles.

b. SEC profiles of SSG_B2 binders.

c. SPR analysis of SSG_B2 binding to recombinant GDF15 proteins. Sensorgrams are shown for analytes ranging from 5 to 100 nM (indicated binder).

d. Sequence alignment of the parental scaffold RGMA with the partial diffused binders (SSG_B1 to SSG_B5). Key hydrophobic (yellow square) and electrostatic (blue square) residues interacting with GDF15 are marked.

e. Complex structural model of SSG_B2 in complex with BMP2, showing electrostatic clashes at the binding interface that are incompatible with productive binding.

f. SPR analysis of SSG_B2 binding to recombinant GDF15 or BMP2 proteins. Sensorgrams are shown for analytes ranging from 5 to 100 nM (indicated binder).

g. Binding interfaces between SSG_B2 and GDF15 site B. Key interacting residues are shown as sticks and labeled.

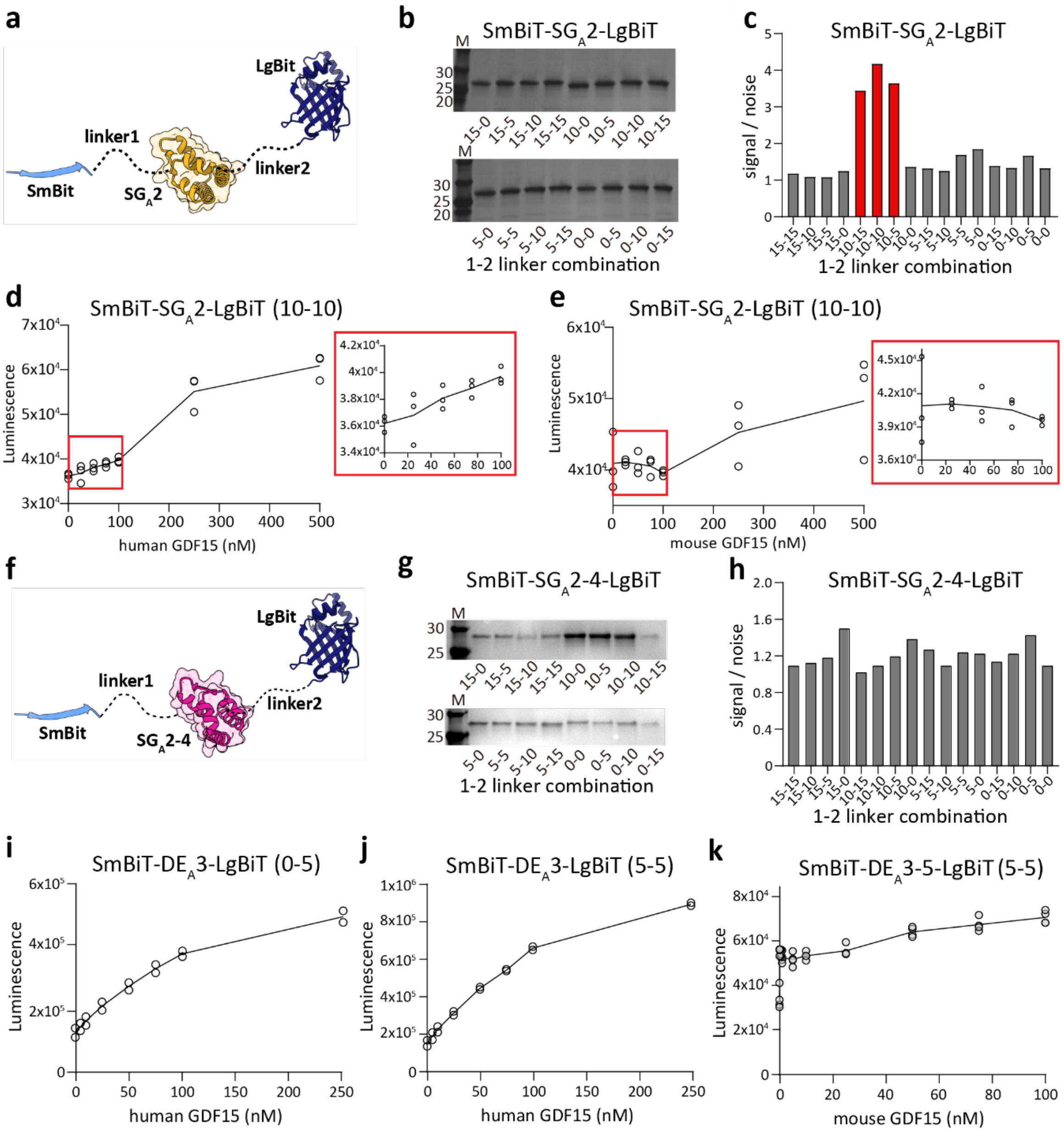

Supplementary Fig. 5. Linker optimization of BAT biosensor with SG$_A$2 and SG$_A$2-derived GDF15 site A binder.

a, f. Structural model of BAT biosensors constructed with SG$_A$2 **(a)** or SG$_A$2-4 **(f)** binders, flanked by SmBiT at the N-terminus and LgBiT at the C-terminus. The lengths of Linker1 and Linker2 were systematically varied to optimize biosensor performance.

b, g. SDS-PAGE analysis of SmBiT–SG$_A$2–LgBiT **(b)** and SmBiT–SG$_A$2-4–LgBiT **(g)** with different linker combinations after *E.coli* expression and affinity purification.

c, h. Screening of linker combinations for SmBiT–SG$_A$2–LgBiT **(c)** and SmBiT–SG$_A$2-4–LgBiT **(h)** by luminescence assay. Signal-to-noise ratios (luminescence intensity of each construct divided by that of the control without GDF15) are plotted, with optimal linker combinations for SG$_A$2 highlighted in red.

d, e. Luminescent signal of SmBiT–SG$_A$2–LgBiT with 10-10 linkers to human GDF15 **(d)** or mouse GDF15 **(e)**. Luminescence (arbitrary units, AU) is plotted against various GDF15 concentrations (n = 3). The linear detection range is indicated with a red box (0–100 nM).

i, j. Luminescent signals of SmBiT–DE$_A$3–LgBiT with 0-5 linkers to human GDF15 **(i)**, with 5-5 linkers to human GDF15 **(j).**

k. Luminescent signals of SmBiT–DE$_A$3-5–LgBiT with 5-5 linkers to mouse GDF15.

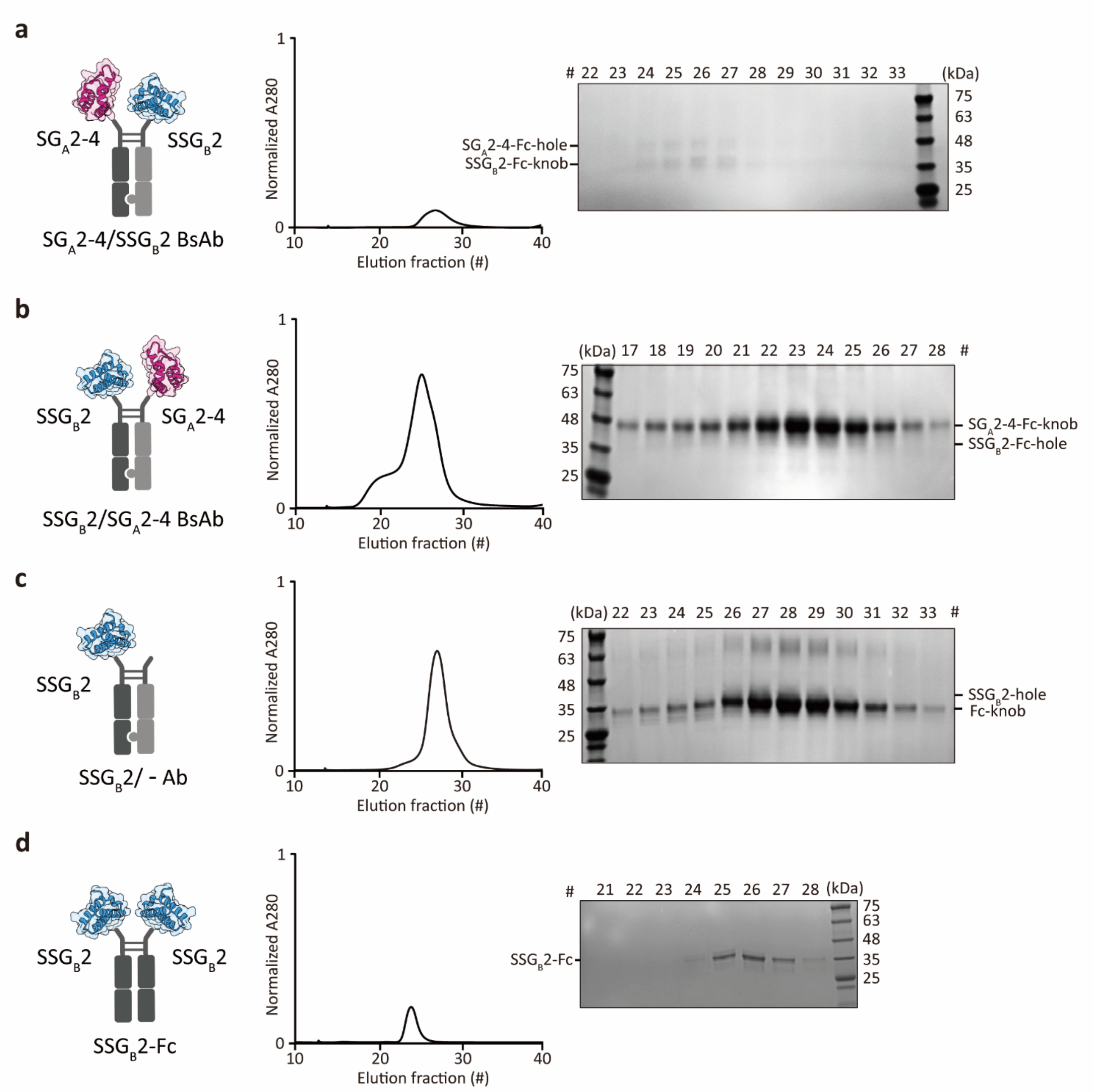

Supplementary Fig. 6. Schematic diagrams and SEC profiles with SDS-PAGE analysis of elution fractions of bispecific SG_A2-4/SSG_B2-Fc (a), SSG_B2/SG_A2-4-Fc (b), SSG_B2/- -Fc (c), and whole SSG_B2-Fc (d).

Supplementary Table. 1. Amino acid sequence of designed GDF15 binders

Binder name	Sequence
**SG_A 1**	TTCLEVAEKLLANPLANEQLAKYLRACSLRGAPCDRETCLAALEDFLKNIPEELRELWLNCSCPPTDKDCRQLKRQLLKLDCL
**SG_A 2**	MTCLEVAEKIVEDPECNAKLAEYLKACSFHGAPCDPEVCKAALEDFEKNIPPELLEAWKNCSCPPTDQDCLQLKKQLKALDCL
**SG_A 2-1**	VSVLDAVEALIADPKSNAALAAHLKAVDALPAGATKAQVDAAYNAFLAALSPELAALVRNAKPDPTDAGERAIYDQLKGTTKL
**SG_A 2-2**	LSWVDALEVVLKDPAENVHLAAYLKAIDALPADVTLEQVQAVYDAFLAKLSPEGQAAITSAKYDPTDPDNNQLYLQVRGLTKV
**SG_A 2-3**	MTVFEALDVVLADPADNAALAEYLKAVRALPPTAPRARVDALYAALLAALSPEAAEALRNATVSPTDPDEQQLYLQLRGLDVL
**SG_A 2-4**	VTVFEALDAIIADPQSNAALAAHLKAVAALPADATKAQVDAIYDAFLAQLPAEVRAKVLAAKPDPTNPDEIQLYNQLRGYDKL
**SG_A 2-5**	ISPIEAAELLIADPKANAALAAHLKAVDALPAGATQAQVDAARDAFLATLTPEQAAAVTNAKPDPTNAGEQAVLAQLLGTTKL
**DE_A 1**	ATAVAEANAAEAVRGFVELVSPEEARREVDAAAKRLAPNPAIAAALRAALEEALA
**DE_A 2**	SVAANFREAALAGAELGASPESIRAAADRTAALVPEEAAEIRAAAEEAVARA
**DE_A 3**	SESERIATAQSIEAVRDYVRLATAAGVPAAEVLAQIEAAAAKFANGAEILAAGRAALAAA
**DE_A 4**	SEELANATLRANARAVAEGIREIADEDPERAAAMAEALEAAYASTPIAAEVAALAAEVRAYARALA
**DE_A 5**	AAHAAAATAANQRATGEDLVALIEANPDPAESRALLAQYRAQMDAAAAKADAATGAAIRAVRAETAARAEAVIAAK
**DE_A 3-1**	SEAERISVEQGAVALRDYVRLATAAGIPAAEIRAALRAVAAKMPGLAPAADEALALVDAL
**DE_A 3-2**	SAADEVAVANGVEAVRDYVRTARALGVPADEILAAVREAAAKMPDPAALVAEAEAELAAA
**DE_A 3-3**	SLADEVAVAQAQVAVADYVALAAALGVPREEIRAQLEAAVARTPDPARVWPVAEAALAAA
**DE_A 3-4**	SLADAVAVANAAEAVADYARLAAAAGIPAAEIEAAIRAAAAKMPDPATVEAAGLAALAAH
**DE_A 3-5**	SHADEVAVENALEAVADYVRLATALGIPAEEILAAIRAAAAKMPDPARVEAVGREALAAA
**DE_A 3-6**	GLADAVAVAQNAVAIRDYVALAAALGVPAEEIRAAAEAAIARSPDPAALRAVLDAALAAA
**DE_A 3-7**	SPADEIALAQNVAAVRDYVRLAKAAGIPEEEIRAAVEAAAAKSPDPAAVRAAGEAALAEA
**SSG_B1**	GSHLERLRDGLLAALTLPPEARRAALAALLAFARAHWPELIKDPEAMAVVGQLRIEARLAGVDLDP
**SSG_B2**	MTLYEELLRRYLAAQRLSPEERQRAIREILELIKKNMSEVVKDPRAMGLAGTVRVEARLLGVDPNI
**SSG_B3**	MTFTQRLQEMYLAAQNAGPEEQKALLKELLAFAKANRGEVVADPMAAGIAGVARVEARLLGLDVSE
**SSG_B4**	KTPLEAVRERYLRALALPPEAFRAALRELIAEMERHKAETFKDPVARGFYGQLHVEARLAGLPTKA
**SSG_B5**	MKIADEITRRLLAAQALGPEARRAALAALLAWAKAHRAEVQKDPMAQAAVGVVRVEARLAGVDVAA
